# Social Behavior and Meningococcal Carriage in British Teenagers

**DOI:** 10.3201/eid1206.051297

**Published:** 2006-06

**Authors:** Jenny MacLennan, George Kafatos, Keith Neal, Nick Andrews, J. Claire Cameron, Richard Roberts, Meirion R. Evans, Kathy Cann, David N. Baxter, Martin C.J. Maiden, James M. Stuart, on behalf of the United Kingdom Meningococcal Carriage Group

**Affiliations:** * University of Oxford, Oxford, United Kingdom;; † Health Protection Agency, London, United Kingdom;; ‡ University of Nottingham, Nottingham, United Kingdom;; § Health Protection Services Scotland, Glasgow, United Kingdom;; ¶ Health Protection Team (North Wales), Mold, United Kingdom;; # Cardiff University, Cardiff, United Kingdom;; ** Thames Valley Local Health Protection Unit, Aylesbury, United Kingdom;; †† St Thomas' Hospital, Stockport, United Kingdom;; ‡‡ Health Protection Agency Southwest, Stonehouse, United Kingdom

**Keywords:** Risk factors, Meningococcus, Carriage, Epidemiology

## Abstract

Understanding predisposing factors for meningococcal carriage may identify targets for public health interventions. Before mass vaccination with meningococcal group C conjugate vaccine began in autumn 1999, we took pharyngeal swabs from ≈14,000 UK teenagers and collected information on potential risk factors. *Neisseria meningitidis* was cultured from 2,319 (16.7%) of 13,919 swabs. In multivariable analysis, attendance at pubs/clubs, intimate kissing, and cigarette smoking were each independently and strongly associated with increased risk for meningococcal carriage (p<0.001). Carriage in those with none of these risk factors was 7.8%, compared to 32.8% in those with all 3. Passive smoking was also linked to higher risk for carriage, but age, sex, social deprivation, home crowding, or school characteristics had little or no effect. Social behavior, rather than age or sex, can explain the higher frequency of meningococcal carriage among teenagers. A ban on smoking in public places may reduce risk for transmission.

Pharyngeal carriage of *Neisseria meningitidis*, however brief, is a prerequisite for invasive meningococcal disease. Highest age-specific disease attack rates are seen in young infants. Another peak of disease that is accompanied by higher frequency of pharyngeal carriage is seen in teenagers (*1*). *N*. *meningitidis* may be cultured from the pharynx in as many as 1 in 4 teenagers (*2*). Male sex (*2**–**5*), cigarette smoking (*6**–**8*), passive exposure to smoke (*8*), pub patronage, discotheque visits (*9*), antimicrobial drug use (*9**,**10*), kissing (*5*), and overcrowding (*11*) have been associated with carriage, and many of these factors are also risk factors for meningococcal disease (*12**–**15*). Outbreaks of meningococcal disease are well documented in educational institutions (*16*), but no data exist on institutional factors that might contribute to carriage and transmission of meningococci. Social deprivation is associated with meningococcal disease (*17*), but whether it is associated with carriage is unknown. Greater knowledge of risk factors for meningococcal carriage may help to identify useful public health interventions.

In 1999, meningococcal group C conjugate vaccine (MenC) was offered to all persons <18 years of age in the United Kingdom (*18*). We identified risk factors for carriage among 14,000 teenagers as an integral part of a large, 3-year, multicenter study to determine the effect of this mass vaccination program on the carriage of meningococci. A reduction in serogroup C carriage after this intervention has already been reported (*19*). The study size gave us high statistical power to investigate the independent effects of risk factors at both individual and school levels.

## Methods

### Study Population

Students from 15 to 19 years of age who were attending school or college full- or part-time (but not at university) were recruited from centers in 8 geographic regions throughout the United Kingdom (Table 1) as previously described (*19*). The study was approved by the Trent MultiCentre Research Ethics Committee. Culture-positive data were not available for the London center in 1999; consequently, this center was not included in the analysis for this study.

**Table 1:** Sample characteristics by study center, UK Meningococcal Carriage Study, 1999

Center	Swabs		No. swabs by student's sex		No. swabs by school year			No. schools visited by type				Plating method
	n	No. analyzed	M	F	12	13	Other	Compre- hensive	Independent/ grammar	Sixth form college	Further education college	
Bangor	972	971	439	532	529	344	94	3	0	0	3	Direct
Cardiff	1,718	1,712	829	883	916	692	102	7	0	1	1	Direct
Glasgow	2,896	2,896	1,317	1,499	1,823	1,073	0	20	2	0	0	Indirect
Nottingham	1,685	1,654	848	806	659	475	489	1	3	5	0	Direct
Oxford	2,398	2,391	1,175	1,216	1,239	822	309	7	0	0	4	Direct
Plymouth	1,394	1,389	688	701	585	437	366	9	4	0	1	Indirect
Stockport	3,011	2,906	1,598	1,408	1,514	1,023	320	0	0	3	0	Indirect
Total	14,074	13,919	6,874	7,045	7,265	4,866	1,680	47	9	9	9	

The local consultant in communicable disease control (public health) asked schools and colleges in their health authority area to participate in the study, with the aim of selecting a sample of schools that broadly represented the social diversity of that population. Each center trained staff to take pharyngeal swabs according to a standard protocol. The swabbing teams visited schools and colleges from October to December for 3 successive years. In year 1 of the study (1999), swabbing took place immediately before MenC vaccination. All students 15–19 years of age in the last 2 school years before university were eligible for the study. After obtaining signed informed consent from the student (or parent/guardian), swabbing teams took a pharyngeal swab, and the student completed a short questionnaire assessing risk factors for carriage. Participants were questioned about age, sex, home postal code, school year, number of persons and rooms in household (to derive persons/room), sharing of bedroom, previous vaccination with meningococcal polysaccharide vaccine, current and recent antimicrobial drug use, active smoking, passive smoking at home, number of days in the last week they had visited a pub or club, and number of people they had intimately kissed in the last week.

Using data from the 1991 census, the postal code of home residence was used to link each person to an electoral ward and its Carstairs deprivation score (*20*) (http://www.mimas.ac.uk). A higher score reflects a greater level of social deprivation.

The following information was requested about schools and colleges: type of establishment, selective or nonselective entry, independent or state funded, single sex or coeducational, day pupils with or without boarders, and school size (small [<200 pupils], medium [200–499 pupils], or large [>500 pupils]).

Here we present the results from the first year of the study; swabs were collected in November and December 1999. The results represent meningococcal carriage just before MenC immunization.

### Laboratory Methods

Swabs were plated onto selective medium either directly or within 6 hours and incubated in CO_2_ at 37°C. Colonies resembling meningococci were identified by conventional tests, and oxidase-positive, gram-negative diplococci were frozen and stored in duplicate as putative meningococci at –70°C. Plates negative after 24 h were reincubated and examined again after 48 h. All isolates from England and Wales were sent to the Meningococcal Reference Laboratory, Manchester, for typing and subtyping. Scottish isolates were sent to the Scottish Pneumococcal and Meningococcal Reference Laboratory, Stobhill, Glasgow. A sample was considered positive if *N*. *meningitidi*s was confirmed by the reference laboratory. The duplicate isolate was examined if a viable *Neisseria* sp. was not obtained from the initial isolate.

### Statistical Methods

Data from questionnaires were entered twice and validated by using Epi Info version 6.0 (*21*). Data inconsistencies were found and corrected when possible, and efforts were made to clarify incomplete dates of birth. Individual-level risk factors for meningococcal carriage were initially analyzed in single-variable models by using logistic regression in the package Stata 8.0 (StataCorp, College Station, TX, USA). School-level risk factors were initially analyzed individually within a multilevel model with students at level 1 and schools at level 2. All risk factors with p<0.1 were then included in a multilevel logistic regression model for a multivariable analysis; again, individual students were at level 1, and schools were at level 2 of the model with explanatory variables at both levels. Center was regarded as having a fixed effect at the school level. A further analysis regarding center as a third level was undertaken but gave similar results, and the 2-level model is presented (the 3-level model is available on request). Each variable included was tested for significance by using the Wald test. Interactions between significant variables were also investigated. The multilevel analysis was carried out in MLwiN (*22*), and the gllamm command (*23*) in Stata 8.0 was used.

## Results

A total of 14,057 swab samples were obtained. Persons were excluded if they were <15 years or >19 years (n = 101) of age, if their age was not known and their attendance in the last 2 school years (n = 16) could not be confirmed, or if their questionnaire was missing (n = 21). A total of 13,919 (99.0%) questionnaires remained for analysis (Table 1). The analysis included 6,874 male students and 7,045 female students from 74 schools or colleges. The overall frequency of carriage was 2,319 (16.7%) of 13,919 students.

### Single Variable Analysis

**Figure 1:**
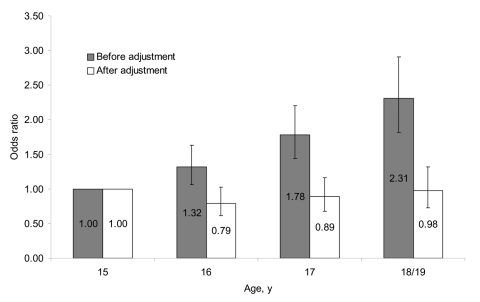
Relationship between age and meningococcal carriage in British teenagers 15–19 years of age before and after adjustment for other factors. Error bars indicate 95% confidence intervals.

Meningococcal carriage increased with age (Table 2, Figure 1). Some social and behavioral factors (level 1 factors) had a strong positive association with meningococcal carriage, namely, cigarette smoking, exposure to passive smoke at home, intimate kissing of >1 persons, and attendance at pubs or clubs in the previous week (Table 2). Weak evidence was found of an association with Carstairs score and number of persons per room. Current or recent antimicrobial drug use was negatively associated with meningococcal carriage. School year was associated with carriage; however, this variable was highly correlated with age and was not considered in the multivariable model. No association was found between carriage and sex, sharing a bedroom, and previous meningococcal polysaccharide vaccination. School-level analysis (Table 3) showed variation in frequency of carriage between centers (7.7%–23.7%, p<0.001). Associations with school type and school size were also highly significant. The presence of boarders, the source of funding, and the gender mix showed no significant association.

**Table 2:** Single-variable analysis of risk factors for meningococcal carriage in British teenagers at an individual level*

Variable	No. swab samples	Total positive (%)	OR (95% CI)	p value
Sex				
Male	6,874	1,156 (16.8)	1.00, reference	
Female	7,045	1,163 (16.5)	0.98 (0.89–1.07)	0.625
Age (y)				
15	959	108 (11.3)	1.00, reference	
16	5,856	839 (14.3)	1.32 (1.06–1.63)	
17	5,575	1,027 (18.4)	1.78 (1.44–2.20)	
18, 19	1,511	342 (22.6)	2.31 (1.82–2.91)	<0.001
School year				
12	7,265	1,096 (15.1)	1.00, reference	
13	4,866	883 (18.1)	1.25 (1.13–1.38)	
Other	1,860	310 (18.5)	1.27 (1.11–1.46)	<0.001
Cigarettes smoked/day				
None	10,732	1,496 (13.9)	1.00, reference	
1–5	1,343	335 (24.9)	2.05 (1.79–2.35)	
6–10	1,016	277 (27.3)	2.31 (1.99–2.68)	
11–20	531	153 (28.8)	2.50 (2.05–3.04)	
>21	46	7 (15.2)	1.11 (0.49–2.48)	<0.001
Other smokers at home				
No	8,457	1,271 (15.0)	1.00, reference	
Yes	5,064	974 (19.2)	1.35 (1.23–1.48)	<0.001
No. persons kissed in last week				
0	7,564	935 (12.4)	1.00, reference	
1	4,910	1,049 (21.4)	1.93 (1.75–2.12)	
2	662	142 (21.5)	1.94 (1.59–2.36)	
3	233	68 (29.2)	2.92 (2.18–3.91)	
4–5	328	79 (24.1)	2.25 (1.73–2.92)	<0.001
No. nights attended pub or club in last week				
0	5,164	523 (10.1)	1.00, reference	
1	3,805	648 (17.0)	1.82 (1.6–2.06)	
2	2,301	482 (20.9)	2.35 (2.05–2.69)	
3	1,207	285 (23.6)	2.74 (2.34–3.22)	
4	562	150 (26.7)	3.23 (2.63–3.98)	
5–7	572	175 (30.6)	3.91 (3.20–4.78)	<0.001
No. persons sharing bedroom				
1	11,900	1,963 (16.5)	1.00, reference	
2	1,662	284 (17.1)	1.04 (0.91–1.20)	
>3	145	22 (15.2)	0.91 (0.57–1.43)	0.751
No. persons/room†				
0–1	13,197	2,196 (16.6)	1.00, reference	
>1–1.5	457	79 (17.3)	1.05 (0.82–1.34)	
>1.5	122	11 (9.0)	0.50 (0.27–0.92)	0.073
Recent antimicrobial drug use				
None	11,749	2,021 (17.2)	1.00, reference	
Current	682	64 (9.4)	0.50 (0.38–0.65)	
Stopped last week	303	48 (15.8)	0.91 (0.66–1.24)	
Stopped last month	733	99 (13.5)	0.75 (0.60–0.93)	<0.001
Prior polysaccharide vaccine				
No	12,493	2,078 (16.6)	1.00, reference	
Yes	1,042	170 (16.3)	0.98 (0.82–1.16)	0.791
Carstairs score, per unit			1.02 (1.00–1.03)	0.022
Month of swabbing				
Nov	7,050	1,192 (16.9)	1.00, reference	
Dec	6,869	1,127 (16.4)	0.96 (0.88–1.05)	0.428

*OR, odds ratio; CI, confidence interval. †Derived data.

**Table 3:** Single variable analysis of risk factors for meningococcal carriage in British teenagers at school level*

Variable	No. schools	% positive†	OR (95% CI)	p value
School type				
Comprehensive	47	13.1	1.00, reference	
Independent/grammar	9	12.0	0.90 (0.60–1.35)	
Sixth form college	9	19.6	1.71 (1.18–2.48)	
Further education college	9	19.1	1.66 (1.14–2.42)	0.002
Funding				
State	68	14.6	1.00, reference	
Independent	6	13.8	0.94 (0.57–1.56)	0.808
Sex		10.5		
Single sex	5	14.8	1.00, reference	
Coeducational	69	14.7	1.52 (0.87–2.64)	0.139
School size				
Small (<200 pupils)	11	13.5	1.00, reference	
Medium (200–499)	47	12.8	0.98 (0.68–1.41)	
Large (>500)	16	20.2	1.78 (1.18–2.69)	<0.001
Boarding				
No	71	14.6	1.00, reference	
Yes	3	13.1	0.79 (0.39–1.61)	0.512
Center				
Cardiff	9	14.2	1.00, reference	
Glasgow	22	11.7	0.75 (0.54–1.03)	
Bangor	6	19.4	1.47 (0.99–2.20)	
Nottingham	9	18.0	1.36 (0.94–1.95)	
Oxford	11	20.8	1.63 (1.16–2.29)	
Plymouth	14	7.9	0.52 (0.36–0.75)	
Stockport	3	23.5	1.92 (1.22–3.03)	<0.001

*OR, odds ratio; CI, confidence interval. †This is calculated as the average of the percentages positive across the schools.

### Multivariable Analysis

In the multivariable analysis, strong associations were found with cigarette smoking, intimate kissing, pub or club patronage, and antimicrobial drug use (Table 4). The association with pub or club attendance showed a clear dose-response relationship. The association with passive smoking was not as strong but remained significant. The rise in carriage by age was much reduced after controlling for these other factors (Figure 1, Table 4). The 15-year-olds had the highest adjusted carriage, but the numbers in this age group were relatively small, and the significant trend in age is attributable to the rise in carriage from 16 years to 18 or 19 years. Associations with Carstairs score and persons per room were no longer significant. Of the level 2 factors, no school characteristics were linked to carriage, and only the association with center remained significant.

**Table 4:** Multivariable analysis of independent risk factors for meningococcal carriage in British teenagers, based on 12,437 samples with complete information*

Variable	OR (95% CI)	p value
Age (y)		
15	1.00, reference	
16	0.79 (0.61–1.03)	
17	0.89 (0.68–1.16)	
18, 19	0.98 (0.73–1.32)	0.025
No. cigarettes smoked/day		
None	1.00, reference	
1–5	1.55 (1.33–1.81)	
6–10	1.69 (1.43–2.00)	
11–20	1.62 (1.29–2.03)	
>21	0.95 (0.41–2.23)	<0.001
Other smokers at home		
No	1.00, reference	
Yes	1.17 (1.05–1.30)	0.004
No. persons kissed in last week		
0	1.00, reference	
1	1.49 (1.34–1.66)	
2	1.25 (1.00–1.57)	
3	2.00 (1.44–2.78)	
4–5	1.41 (1.05–1.91)	<0.001
Nights attended pub or club in last week		
0	1.00, reference	
1	1.52 (1.33–1.75)	
2	1.68 (1.44–1.96)	
3	1.84 (1.52–2.21)	
4	1.90 (1.50–2.42)	
5–7	2.27 (1.79–2.87)	<0.001
No. persons/room		
0–1	1.00, reference	
>1–1.5	1.01 (0.76–1.34)	
>1.5	0.57 (0.29–1.12)	0.267
Recent antimicrobial drug		
None	1.00, reference	
Current	0.51 (0.38–0.67)	
Stopped last week	0.81 (0.57–1.13)	
Stopped last month	0.66 (0.52–0.83)	<0.001
Carstairs score, per unit	1.00 (0.98–1.02)	0.909
School type		
Comprehensive	1.00, reference	
Independent/grammar	1.04 (0.67–1.60)	
Sixth college	0.67 (0.32–1.40)	
College	0.82 (0.49–1.37)	0.681
School size		
Small (<200 pupils)	1.00, reference	
Medium (200–499)	0.92 (0.65–1.30)	
Large (>500)	1.18 (0.6–2.06)	0.617
Center		
Cardiff	1.00, reference	
Glasgow	0.89 (0.62–1.28)	
Bangor	1.33 (0.83–2.14)	
Nottingham	1.45 (0.86–2.44)	
Oxford	1.46 (0.99–2.16)	
Plymouth	0.48 (0.31–0.74)	
Stockport	1.99 (1.01–3.90)	<0.001

*OR, odds ratio; CI, confidence interval.

**Figure 2:**
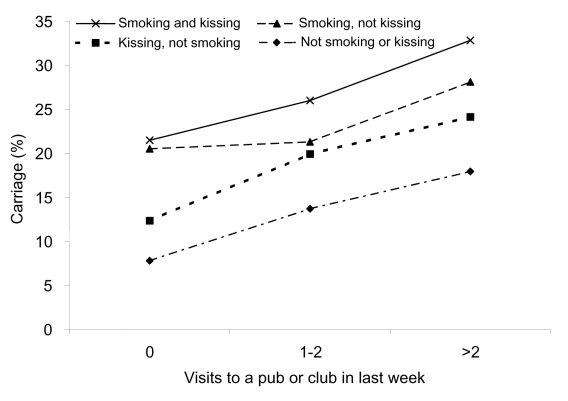
The combined effect of varying attendance at pubs and clubs, cigarette smoking, and intimate kissing on the risk for meningococcal carriage in British teenagers.

The analysis of interactions showed evidence of small, but significant interactions between smoking and kissing (p = 0.005) and also between smoking and pub or club attendance (p = 0.003). Investigation of the relationship between these 3 variables showed that crude carriage rates varied from 7.8% to 32.8% (Figure 2). The interaction effect appears to be due to relatively high carriage (20.5%) in teenagers whose only risk factor among these 3 is smoking. This analysis was repeated by calculating odds ratios from the multivariable analysis for all combinations of the 3 variables, and the pattern was similar to that seen with the crude carriage rates.

## Discussion

To our knowledge, this is the largest study, several times larger than other published studies (*2**–**10*), that examines risk factors for meningococcal carriage. Humans are the only natural hosts for meningococcus, and carriage in the nasopharynx, however brief, is both a prerequisite of invasive disease and essential for transmission. Our study strongly suggests that behavior, not age, is largely responsible for the increase in meningococcal carriage seen in teenagers. Active and passive smoking, intimate kissing, and attending pubs and clubs were all strongly and independently linked to the risk for meningococcal carriage. The size of this study allowed us to quantify these contributions and identify a "dose-dependent" increase in risk for attendance at pubs and clubs. The presence of all 3 risk factors increased the risk of carriage 4-fold, compared to the baseline risk in teenagers with none of these factors. The results of the risk factor analysis in the succeeding study years (2000 and 2001) were similar to those presented here. The same factors were significant, and a dose-response relationship to smoking was found in 2000.

Active and passive smoking have both been linked to risk for meningococcal carriage (*6*). Both are shown as independent risk factors in this study, and the increasing risk with the number of days that persons visited pubs or clubs may well be due to passive smoking. Other possible risk factors associated with pub and club attendance include alcohol consumption (*24*) and overcrowding (*11*). Loud music may indirectly increase risk for transmission as persons raise their voices and move closer to each other to be heard. Although salivary contact itself is probably not a risk factor (*25*), frequency of intimate kissing would be expected to increase risk for transmission through close contact with respiratory droplets from the nasopharynx (*14*).

The overall prevalence of carriage was close to expected levels for a European population of this age group, mainly 16- to 17-year-olds (*2**,**3*). Although increasing age showed a strong relationship with increasing prevalence of carriage in the univariable analysis, this increase was much reduced after adjustment for other factors. This observation is striking since other studies have suggested an increased risk of meningococcal carriage with age (*2**–**5*). We observed no association of sex with carriage, in contrast to results of other studies (*2**–**5*). This finding strongly suggests that behavior, rather than age or sex, is the driving force behind the increased risk of meningococcal carriage in teenagers. This study involved ≈14,000 persons, had more power than those previously undertaken, and was restricted to older teenagers.

This study is the first to examine a link between meningococcal carriage and social deprivation in the United Kingdom. We found no evidence of an association. The methods used had some limitations, since we were only able to link by postal code to a ward and not to individual households. These findings contrast with the increased risk for disease in young children found in lower socioeconomic groups by using similar methods (*26**–**28*). However, this association has only been reported in young children and may not apply to the teenage population. Some previous studies have reported an association between crowding and meningococcal carriage (*11**,**29*) and meningococcal disease (*13**,**30*). This study showed no evidence that increasing levels of crowding in the home, as measured by the number of rooms or persons per household and number of persons per room, was associated with increasing levels of meningococcal carriage. Crowding in the home may also be less relevant to teenagers than to young children because teenagers spend less time at home.

Of the study participants, 16% reported previous meningococcal vaccination. The only meningococcal vaccine available before this study was the plain polysaccharide vaccine. The lack of impact of this vaccine on carriage is not surprising. Any effect of polysaccharide vaccination on carriage is probably short term, and the most commonly used polysaccharide vaccine is directed against serogroups A and C. Very few carriers of serogroup A and C strains were found in this study. A protective effect from recent antimicrobial drug use was expected because many antimicrobial drugs temporarily suppress or eradicate meningococcal carriage (*31*).

The design and size of this study allowed us to examine school characteristics as possible risk factors for meningococcal carriage. Although outbreaks often occur in educational institutions, no previous data existed on institutional factors that might contribute to carriage and transmission of meningococci. We did not identify any school characteristics that had an independent effect on carriage. Differences between centers remained significant even after adjustment for other factors. These may have been true differences between centers or the result of differences in the methods of swabbing, plating, and laboratory procedures. For example, in 1 study, direct plating resulted in a doubling of the detectable frequency of carriage, compared to results of indirect plating (*32*).

In conclusion, this study suggests that the rise in meningococcal carriage in teenagers is driven by changes in social behavior. Since carriage is a prerequisite for invasive disease (*33*), this rise in carriage is likely to explain the well-documented peak in meningococcal disease attack rates in teenagers (*1**,**34**–**36*). Explaining the risks of smoking, intimate kissing, and pub and club attendance may be a useful public health intervention, particularly in an outbreak situation. In the United Kingdom, a ban on smoking in public places will be introduced in 2007 (*37*). Potential health benefits from such a measure may include a reduction in the risk of meningococcal meningitis and septicemia.
